# The nature of holistic processing in face and object recognition: current opinions

**DOI:** 10.3389/fpsyg.2014.00003

**Published:** 2014-01-24

**Authors:** Tamara L. Watson, Rachel A. Robbins

**Affiliations:** Foundational Processes of Behaviour Research Laboratories, School of Social Science and Psychology, University of Western SydneySydney, NSW, Australia

**Keywords:** face recognition, holistic processing, configural processing, inversion effect, other-race effect

Despite serious advances in our understanding of how we cognitively and neurally achieve the amazing range of perceptual decisions we can make from faces, the picture is not complete. Recently, exciting developments have been made in terms of new questions to pose, new directions from which to approach existing questions, new research methods and insights from looking back and taking stock.

Each approach contained in this special issue is attempting to re-think what we know about holistic or configural face recognition. While it is clear that faces are of prime importance to humans, we have not been able to answer what is meant by configural or holistic processing. In general, it is defined as something like integration across the area of the face, or processing of the relationships between features as well as, or instead of, the features themselves. The methods, questions and thought provoking commentary contained in this issue represents the new wave of attempts to better define and outline how we process faces.

The papers range from brain imaging and eye-tracking, through to special populations, cross-race differences, the evolutionary nature of holistic processing and computational models. Below we summarize the articles included and present some thoughts about the implications of these papers for the field.

This Research Topic includes four reviews, each taking a slightly different approach. Piepers and Robbins (2012) provide a historical review of terms and common measures and suggest important advances can be made by studying moving faces, helping us better understand whether “relationships between parts” means ‘between the edges of nameable features’ or ‘the center of key elements.’ Richler et al. (2012) argue that much of the confusion in the area is caused by a lack of one-to-one mapping between theoretical constructs and measured effects, and review how they think some of these measures and meanings may relate. Taking a different approach, Burke and Sulikowski (2013) review possible holistic processing in non-human animals. Importantly this review also notes that the question of whether holistic processing is an innate face system or an expertise mechanism co-opted for faces is not really relevant to whether there is an evolved mechanism. Watson (2013) reviews holistic processing as it relates to two special populations—those with autism and schizophrenia. She concludes that our experimental tasks have not been sensitive enough to elucidate the changes to face recognition in these populations.

Four papers provided data on basic aspects of holistic processing. Favelle and Palmisano (2012) tested inversion effects of faces rotated in yaw (left-right changes in view) and pitch (forward-backward head tilt). They suggest that holistic representations of faces may not include extreme views. Vesker and Wilson (2013) assess the relationship between the eyes (interocular separation) and show that Vernier type reference to other individual features can look like a holistic representation. They therefore suggest that nameable features are not the code of configural processing, but rather a series of edge points. Mestry et al. (2012) introduce the use of measures of sensitivity and bias with probit regression models. Using this method they uncover perceptual effects that previous studies have attributed to purely decisional factors. Finally, McKone et al. (2013) suggest that inverted control conditions should always be tested for the composite and part-whole task. They present data on race, sex and half-to-match tasks to illustrate that factors other than holistic processing may influence the results.

Two papers examined cross-race effects in face recognition. Crookes et al. (2013) show that Chinese participants can process all faces holistically and that this is not a general object recognition strategy. Miellet et al. (2013) used eye-tracking with cross race participants and highlight that both part-based and holistic processing of faces is important, but culture as well as aspects like task may influence which is used.

Goffaux et al. (2013) use fMRI to look at the functional role of the FFA depending on the task at hand and suggest that the FFA does both local and “holistic” processing, identifying a responsiveness/flexibility of function.

Looking at face detection, Paras and Webster (2013) reveal that important factors to seeing faces in visual noise are symmetry and having some dark eye-like features with a roughly face-like configuration of blobs. However, they note that such “faces” differ from actual faces in fixations, and do not elicit a N170 in ERP (as actual faces do).

Wallis (2013) provides a computational model to produce insight into how a neurally inspired system might be organized to provide face recognition like abilities using a set of neural tools that are common to object recognition.

While this collection of work has not provided a definitive answer it should provide some inspiration as the foundation for new and exciting directions in this field that will make an enormous contribution. This is the beginning of the new wave of face research; watch out for these authors' contributions in the future as we move forward in leaps and bounds.
